# The Impact of Perceived Risk on Consumers’ Cross-Platform Buying Behavior

**DOI:** 10.3389/fpsyg.2020.592246

**Published:** 2020-10-29

**Authors:** Xiaoxue Zhang, Xiaofeng Yu

**Affiliations:** School of Media and Communication, Shenzhen University, Shenzhen, China

**Keywords:** perceived risk, trust, negative reports, consumer cross-platform buying behavior, Xiaohongshu

## Abstract

In recent years, social application with shopping has become an indispensable activity in people’s daily lives. A number of previous studies have investigated various risks in online shopping and consumer’s belief. However, few scholars paid more attention to buying behavior, especially on consumer’s cross-platform buying behavior. Although consumers can purchase goods directly in platforms that have given them information, they also buy target products on other platforms. This study conceptualizes and implements concepts such as perceived risk, trust, negative reports, and consumer cross-platform buying behavior. A questionnaire survey of users (*n* = 366) was conducted, and a basic description and comparison of consumers’ cross-platform buying behavior were made in terms of consumers’ perception of the risk of product effects, consumers’ perception of service risks, and consumers’ perceptions of other risks. The degree of trust in the platform and negative reports about the platform also affect cross-platform buying behavior. As a result, these findings are discussed and explained, and some reference is provided for related platforms. Besides, the study’s finding concerning consumer’s buying behavior depends on the security of platform in people’s conceptions.

## Introduction

Mobile devices have become one of the most popular and ubiquitous technological devices around the world (Shiau et al., 2017). According to the 43rd Statistical Report on Internet Development in China released by the China Internet Network Information Center (CNNIC) in Beijing, as of December 2018, the number of mobile phone Internet users in China reached 817 million, and the proportion of users accessing the Internet through mobile phones reached 98.6%. The number of mobile phone network payment users in China reached 587 million, with an annual growth rate of 10.7%. Besides, the use rate of mobile phone Internet users reached 71.4%. The proportion of netizens paying *via* mobile networks when spending offline also increased from 65.5% at the end of 2017 to 67.2% (China Internet Network Information Center, 2019). It can be seen from the data that with the developments of the times, payment habits have gradually changed on a large scale (Świecka et al., 2020). Shopping habits have also gradually shifted from offline to online, but even and when shopping offline, mobile payment has gradually become people’s consumption payment habit.

The advancement of Internet changes people’s consumption behavior remarkbly. Since 2011, social e-commerce platforms such as Xiaohongshu, Mogujie, and Meilishuo have sprung up (Zhao et al., 2020). This type of platform employs user-generated content as a key tool to assist online shoppers (Zhu, 2014). Electronic word of mouth (eWOM) has become an important part of social commerce (Zhao et al., 2020). And in this process, these applications play an important role in their decisions. When searching for information on the Internet, it is quite possible that consumers consult a social media website including Facebook and Twitter (Giustini, 2006), rather than a more traditional website of an official information body (Rutsaert et al., 2013a,b). Social networks are not only used to keep in touch with others; they have been applied to a wide range of purposes and have exerted an increasing impact on society (Shiau et al., 2018). As long as people have a smart phone, they can buy most of the things that they want to buy. Internet use for information searches, shopping, and leisure is growing (Armstrong et al., 2000; Ceyhan, 2010). Also, it has changed into a dynamic information environment of social media, where almost anyone can post messages and spread or comment on information rapidly (Horst et al., 2007). Nowadays, with the advancement of technology and the continued pressure to produce and consume amid resource scarcities, both consumers and organizations are increasingly embracing the idea to collaborate and share in producing and consuming marketing exchanges in the marketplace (Chen and Wang, 2019). People are more likely to recommend to a larger circle of people when faced with utilitarian products, affective messages, and active seller participation than when faced with hedonic products, rational messages, and passive seller participation (Lim, 2017a).

Social media also empower consumers to interact with other consumers and express their own opinion (Shao, 2009), resulting in an increase of public involvement and interaction (Rutsaert et al., 2013b). The “Click-and-Tell” feature leveraged the word-of-mouth effect and let potential buyers recruit new customers (Dai and Kauffman, 2002). eWOM has a positive effect on consumers’ perceived value (PV) and perceived risk (PR) of using online group buying (OGB) sites, which in turn have a significant influence on their intentions to shop at OGB sites (Lim, 2015). And it will motivate the sense of virtual community. The sense of virtual community and perceived critical mass represent social influences that have a significant effect on consumers’ behavioral intentions to engage in OGB. Sense of virtual community exerts a significant influence on perceived risk, which in turn is significantly related to OGB intention (Lim, 2014). At the same time, Lim (2017b) examined the link among consumer characteristics (price-sensitive behavior, variety-seeking behavior, and compulsive buying behavior), shopping values (utilitarian and hedonic shopping values), and behavioral intention in OGB. However, online shopping is a double-edged sword. When people buy their favorite products, there can still be a series of problems. For example, a poor understanding of product attributes makes consumers buy useless things or buy things inconsistent with the goods displayed by the merchant. Often, these phenomena make consumers want to buy some products, but sometimes there are not enough user reviews to be used as a reference, resulting in people’s demands for obtaining effective information about products becoming increasingly important. And then they will have their own sales channel to sell something to users. However, users tend to buy these products in some platforms they often purchase. The study is driven by one main research question: What influence do consumers buy production in other platforms rather than the platform that they acquire information?

The vital contributions of the study are as follows: The authors offer a theoretical framework through which various factors might affect consumers’ cross-platform purchase behavior more effectively. This study analyses the relationship between the perceived products risk, perceived service risk, trust from other platforms, negative report, and consumers’ cross-platform purchase behavior. The results are helpful to develop a consumer online shopping decisions theory based on applications especially social media or online shopping application. At the same time, this study helps some enterprises to have better service and public relations.

## Literature Review

The theoretical framework in the study is primarily the theories of perceived risk and trust, which further explores consumer cross-platform buying behaviors in conjunction with negative platform reports.

### Perceived Risk

With the increase of network users, social network developers must consider the social factors that influence the intention to use social networks (Cheung et al., 2011). For instance, how the perceived usefulness (or performance expectancy) and perceived ease of use (or effort expectancy) have an effect on the continual use of information technology (IT) (Davis, 1989; Rana et al., 2016, 2017; Dwivedi et al., 2017a,b). When it is possible for a loss to occur, people often exhibit a contrasting reaction called the reflection effect (Kauffman et al., 2010).

Perceived risk has always been an important content for academic research. The variables of reader’s motivational involvement, such as experience, prior knowledge, perceived risk, and information need were measured through paradigms as developed by Wu and Shaffer (1987); Hennig-Thurau et al. (2004), Huang et al. (2009); Jeong and Jang (2011), Martin et al. (2015); Hussain et al. (2017), and Yang (2017). Consumers’ risk perception with respect to the network also relates to their risk perception for specific apps. At the same time, it represents consumer uncertainly about loss or gain in a particular transaction (Murray, 1991). Customers form service expectations according to their past experiences, word of mouth, and advertisements; service quality is used to assess and compare perceived and expected services (Shiau and Chau, 2016). And a quantitative study was conducted based on the technology acceptance model (TAM),and indicated that perceived usefulness, perceived ease of use, and perceived risk all have a significant relationship with consumer attitudes, which subsequently have a significant effect on intention to use OGB sites (Lim, 2014). The significance of perceived sacrifice, perceived risk, perceived benefit, and perceived quality on consumer perceptual evaluations of purchase equity in OGB (Lim, 2018).

The research results of perceived risk in this field are extremely fruitful. Perceived risks refer to the spirit cost associated with customers’ purchasing behavior, which represents a kind of uncertainty about the future. This uncertainty will directly affect the consumers’ purchase intention (Wei et al., 2018, p. 4). Bauer (1960) defined perceived risk as the risk that consumers actively perceive because they do not understand product information. Later, Bauer introduced perceived risk to consumer behavioral analysis. In the 21st century, scholars also began to pay attention to the perceived risk of online shopping. Moreover, users can access these services through various mobile devices at different times and in different contexts of the interaction (Pernici and Krogstie, 2006). In the information age, it plays an important role in people’s lives. It is because people have much more right to choose different applications and services to satisfy their needs. The perceived risk of online shopping is a kind of loss for consumers in online shopping, which is subjective expectation (Forsythe and Shi, 2003, p. 869). From the above definition of perceived risk, it can be clearly understood that consumer perception of risk is an inner experience that cannot be observed directly; the dimensions of risk can only be inferred by certain indicators. Consumers consequently have to make up their minds regarding purchasing and consuming those products (Hilverda et al., 2018, p. 3). And the importance of service quality has been stressed in the information system field because of the increasing number and type of services delivered using websites (Cenfetelli et al., 2008; Xu et al., 2013).

Risk attitudes can be quantified along a continuum from risk-averse to risk-seeking (Kauffman and Wang, 2001). Although the perceived risk can be divided into many dimensions, and these dimensions are measured through various indicators, to make an appropriate definition, the relevant risk dimensions are considered according to the research direction, research scenario, and research method being used. According to the authors’ current research, and by reading the literature and combining the shopping situation for Xiaohongshu and the overall grasp of this research, the two important dimensions of risk, with respect to consumer perceptions, are being taken as the perceived product effect risk and perceived service risk in online shopping on the app. Because of the advancement of Internet technology (IT), assessing service quality is critical in the relatively new domain of online business, in which firms deliver products and services through web channels (Shiau and Chau, 2016). It is because that IT provides the medium for delivering the service (Gefen, 2002). Compared to the service, A type of product that is tangible, given that goods are typically manufactured, stored, transported, marketed, and sold (Zeithaml et al., 1985; Gadrey, 2000). This is contrary to services, which is an intangible product, because it encapsulates the behavior of doing something for someone or something (Ballantyne and Varey, 2008; Vargo and Lusch, 2008).

Advantages of using social media to disseminate information are, for instance, speed and accessibility (Rutsaert et al., 2014). In addition, the perceived product effect risk refers to consumers’ concerns that products purchased through the Internet may be poor in terms of quality and performance and may not achieve the expected results. As a result, the authors define “consumer perception of product risk” as in the online shopping environment, the perceived product effect risk caused by the inability to judge product quality and performance through touch, direct feeling, or trial (Forsythe and Shi, 2003). Also, the authors define “consumer perception of service risks” as after-sales service risk and delivery service risk. And in this study, the authors define “consumer cross-platform buying behavior” as consumers only search for useful information about the target product on certain types of apps, with product notes and shopping functions as one, and they do not or rarely trigger the final purchase behavior on such apps. On the contrary, they often make purchases on other apps.

Based on this, the authors pay attention to the importance of consumer perception of product risk and consumer perception of service risks in the process of purchase and make the following assumptions:

H1: The risk perception of product effects for consumers has a positive impact on the cross-platform purchase behavior.
H2: The risk perception of services for consumers has a positive impact on the cross-platform purchase behavior.

### Trust

Mobile commerce is one of the research topics that are heavily involved in the application of Mobile Information Systems. Because new technologies usher in many market opportunities, scientists have concentrated on different levels of framework for mobile commerce (Shiau et al., 2019). In online shopping, trust is one of the most important factors for scholars to consider. Shiau’s study focuses on the group buying online and considers trust as a key factor. In his study, he used the application of social exchange theory (SET) in the group buying context and identified the issue in the context of OGB by incorporating factors such as reciprocity, reputation, trust, satisfaction, and seller creativity (Shiau and Luo, 2012). And then they examined the effects of altruistic and egotistic behavior on online group repurchasing intention through the psychological processes of trust and satisfaction (Shiau and Chau, 2013).

Trust is a kind of belief, that is, it feels that the other side will take actions to realize the interests of one’s own side and try to reduce the harm to one’s own side because of its long-term dependence on one’s own (Anderson, 1990). Trust is the confidence that consumers have in an organization or e-seller in an online context (Shiau and Luo, 2012).

Some propose a broad definition of trust as an undefended mental state based on a positive estimation of the intention and behavior of the other party (Rousseau et al., 1998). In a broad sense, trust is the willingness of a party to be vulnerable to the actions of another based on expectations that the other has positive intentions and actions toward the one trusting (Mayer et al., 1995; Rousseau et al., 1998; Malhotra and Lumineau, 2011; Stern and Coleman, 2015; Riley et al., 2018). Therefore, an unguarded mental state is one of the important elements of trust. Defense is a kind of intention of relying on exchange partners and being willing to take risks. As a result, the definition of trust as a psychological state also allows for institutions to be targets of trust (PytlikZillig and Kimbrough, 2016). In regard to agency or inter-organizational collaborations, trust has been described as the “willingness to rely on an exchange partner in whom one has confidence” (Ganesan, 1994). Because of those reasons, trust is also a key determinant in the adoption of new IT (Gefen, 2004) of many kinds, including e-commerce (Gefen et al., 2003), virtual teams (Jarvenpaa et al., 1998), online communities (Ridings et al., 2002), online software marketplaces (Gefen and Carmel, 2008), online consumer marketplaces such as eBay (Pavlou and Gefen, 2004, 2005; Pavlou and Fygenson, 2006), e-banking (Kaabachi et al., 2017; Ofori et al., 2017), and e-government (Warkentin et al., 2018), among others.

The trust in this article is based on consumers and the merchants behind the Xiaohongshu platform. There is no doubt that this is an association with unfamiliar people. The degree of consumers’ trust is defined for other platforms (Taobao, Jingdong, Haitao, or acquaintances), when conducting cross-platform purchases, as an existence that is used to maintain the relationship between individuals or between individuals and platforms.

The relationship between perceived risk and trust has always been an issue of particular concern to the academic community, which have different views on the relationship between the two. One view is that perceived risk and trust independently affect behavior, and there is no correlation between the two; a second view is that perceived risk is a prerequisite for trust. Most of the studies on the relationship between perceived risk and trust use empirical research methods. The method of explaining the structural model not only proposes a hierarchical relationship that affects user trust but also provides theoretical support for the relationship between perceived risk and trust. This study will validate the second major kind of assumption, which is that perceived risk is a prerequisite for trust. Based on this, the authors make the following assumptions:

H3: Perceived risk of product effects will affect consumers’ trust in other platforms and will further affect consumers’ cross-platform buying behavior.
H4: Perceived service risk will affect consumers’ trust in other platforms and will further affect consumers’ cross-platform buying behavior.

### Negative Reports

With the development of modern society, it means that media use is changing each day. In a society featuring globalization, social networks span spatial and temporal restrictions and satisfy demands for interpersonal interactions at any time and at any place (Wellman et al., 2001). Several studies have demonstrated the impact of positive and negative online reactions of other consumers on behavior (Winterbottom et al., 2008) and attitudes (Vermeulen and Seegers, 2009). For instance, some people employed Xiaohongshu app to convey some obscene information, giving rise to the lower frame of Xiaohongshu. The channels by which this group contacts media mainly include media reports obtained through the Internet. Such news reports can be divided into positive reports, neutral reports, and negative reports based on the mood of the text and the tone of the words.

Negative media reports have a negative impact on companies’ performance. If the report content is serious, the impact will be aggravated; this study defines negative reports as those reports that include derogatory terms such as disclosure, criticism, and questioning or include exposure of news that is not conducive to the sustainable development of the platform, as well as an in-depth analysis of the company’s potential problems. The authors define “negative report” as this will mainly include reports that include derogatory terms such as disclosure, criticism, and questioning or that expose some news that are not conducive to the sustainable development of the platform, as well as the type of report that deeply analyses the company’s potential problems. Considering the role and impact of negative reporting on consumers’ cross-platform, the authors put forward the following assumptions:

H5: Perceived risk of product effect will affect consumers’ trust in other platforms and will further affect consumers’ cross-platform buying behavior. However, this influence is positive when there are many negative reports and negative when there are few negative reports.
H6: Perceived service risk will affect consumers’ trust in other platforms, cross-platform further affects consumer buying behavior. However, this influence is positive when there are many negative reports and negative when there are few negative reports.

**FIGURE 1:**
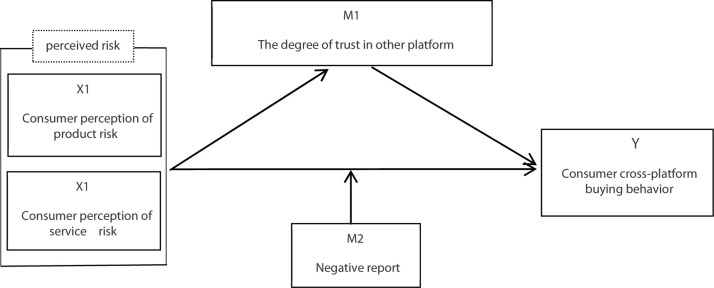
Theoretical model.

Finally, considering the particularity of the Xiaohongshu app and related research at home and abroad, the authors have constructed a theoretical model of “perceived risk → consumer cross-platform buying behavior.” This model was used to conduct an empirical analysis to explore the impact of social sharing on consumers’ cross-platform buying behavior. The model is shown Figure 1.

## Research Method

### Context

The Xiaohongshu application integrates strategy and shopping, and it is one of the largest social business platforms in China, and it is also a community-based e-commerce platform. And it was created as a sharing platform “fulishe” of two modules with the joint manipulation of operations. The group targeted by Xiaohongshu includes the post-1990s and post-2000s user groups. Generally, such platforms are likely to be relevant when the distance between sharing communities is close (e.g., local neighborhoods) and when the goal is to improve the connectivity between local communities (e.g., farming estates and rural villages) (Lim, 2020). The Xiaohongshu application makes the best use of the strength of community. Initially, Xiaohongshu could only be shared as a community platform for overseas travel and original shoppers, but it gradually opened up users’ shopping notes in the market and accumulated many seed users in this market. These users have provided sufficient information about products for the “fulishe” of the Xiaohongshu app’s e-commerce sector. Moreover, they have a positive effect on people’s sense of happiness and enhance their participation in community and social activities (Sheldon, 2008).

Innovation is one of the most critical forces in creating new services and products, developing new markets, promoting organizations’ competitiveness, and transforming industries (Hameed et al., 2012). The most important factor is Xiaohongshu app’s closed-loop operations. The Xiaohongshu app’s main loop is formed by the five elements of the ecological chain, including information, services, payment, data, and logistics, while the closed-loop physical process and word of mouth are the main closed-loop operation model. And it is a cross-border electricity supplier application that can share some useful stuff with others. At the same time, everyone can share their shopping experience and moods with others. In ambiguous situations where consumers are uncertain about an appropriate course of action, they may adjust to the viewpoints and behaviors of others who they assume to be more knowledgeable in dealing with the particular situation (Hilverda et al., 2018, p. 2). It means that consumers can use the Internet anytime and anywhere to collect, pay, and share information about purchasing decisions (McKinsey and Company, 2019). There is no doubt that Xiaohongshu becomes more and more popular among youngsters in China. Product experiences are posted on the platform to give consumers a certain reference meaning, so they can avoid buying any wrong products as much as possible. Xiaohongshu is a set of community-based electronic business platforms in China, created as a sharing platform “fulishe” of two modules with the joint manipulation of operations. Initially, Xiaohongshu could only be shared as a community platform for overseas travel and original shoppers, but it gradually opened up users’ shopping notes in the market and accumulated many seed users in this market. These users have provided sufficient nutrition for the “fulishe” of the Xiaohongshu app’s e-commerce sector, so this “fulishe” can earn 300 million yuan in sales without any advertising. The most important factor is Xiaohongshu app’s closed-loop operations. The Xiaohongshu app’s main loop is formed by the five elements of the ecological chain, including information, services, payment, data, and logistics, while the closed-loop physical process and word of mouth are the main closed-loop operation model.

### Questionnaire Design

The questionnaire for this research includes two parts. The first part is the survey of consumers’ perceived risks on consumers’ cross-platform purchasing behavior, including perceived product effect risks, service risks, and other platforms (such as Taobao, Jingdong, Haitao, and Acquaintance purchasing), as well as the four parts of trust and negative reporting. The second part is a survey of consumers’ basic situation. A questionnaire survey is the main research method used in this study. The authors combed through a considerable amount of domestic and foreign literature on perceived risk, trust, and consumer purchasing behavior. Based on previous research, in view of the actual situation with the Xiaohongshu app, the existing research results were finally designed for this study.

A Likert five-level scale is the main component of the questionnaire in this study. There are three or four sets of items on the measurement content in different scales, and each group of question item statements is measured by scoring. The scoring items are divided into five items: points for strong disagreement, points for disagreement, points for uncertainty, points for agreement, and points for strong agreement. The continuous-variable measurement scale and its sources are shown in Table 1.

**TABLE 1:** Variable measurement scale.

**Variable**	**Code**	**Measuring project**
Consumer perception of product risk	A1	I think there is a quality problem with Xiaohongshu’s goods.
	A2	I don’t think the use effect of the goods in Xiaohongshu meets my requirements.
	A3	I think there are fake and inferior products in Xiaohongshu.
	A4	I think there is a safety hazard in Xiaohongshu’s products.
Consumer perception of service risk	B1	I think the customer service staff of Xiaohongshu reply efficiency is low.
	B2	I don’t think Xiaohongshu can provide the necessary after-sales service and perfect return and replacement service quickly.
	B3	I think Xiaohongshu’s imperfect logistics distribution system may lead to the failure to receive the goods on time.
The degree of trust in other platforms (Taobao, Jingdong, Haitao, etc.)	C1	Compared with Xiaohongshu, I am more familiar with other shopping apps like Taobao and Jingdong.
	C2	Compared with Xiaohongshu, I think the products from other shopping apps like Taobao and Jingdong are more trustworthy.
	C3	Compared with Xiaohongshu, I have more confidence in the future of other apps like Taobao and Jingdong.
Negative report	D1	I think Xiaohongshu sometimes has a negative impact.
	D2	I think the level of negative news about Xiaohongshu is high.
	D3	The negative coverage of Xiaohongshu will affect my use of Xiaohongshu.
Consumer cross-platform buying behavior	E1	I think Xiaohongshu is more of an information-sharing platform than a shopping platform.
	E2	Compared with purchasing products on Xiaohongshu, the authors prefer to purchase target products searched on Xiaohongshu through other platforms.
	E3	I will search for useful information on my favorite products in Xiaohongshu for many times and then purchase the products I find through other platforms.

The questionnaire in this study includes two parts. The first part is the basic usage of Xiaohongshu by consumers, which involves whether to use Xiaohongshu, the frequency of shopping on Xiaohongshu and how Xiaohongshu is used. It also involves the search for the price of a single product and the main category of the search on Xiaohongshu. The second part is the main part of the questionnaire. A total of 16 questions are designed to measure Xiaohongshu’s perceived product effect risk, perceived service risk, and trust level with respect to other platforms (such as Taobao, Jingdong, Haitao, and acquaintance purchasing), as well as negative reporting and consumer cross-platform buying behavior.

This study was conducted in October 2019, and the authors chose a smaller sample size to do a pre-test of the questionnaire by Wen Juanxing (an online service for questionnaire). A total of 68 test questionnaires were distributed online. The sample test results showed that five of the questionnaires were invalid, and there were 63 valid copies. After SPSS 25.0 analysis, the reliability and validity tests were all within the ideal range. Therefore, the authors finalized the questionnaire for this study. This questionnaire survey used the same platform – Wen Juanxing. Before beginning to partake in this study, participants were given information explaining the aim of the study. After providing informed consent, they were allowed to proceed. The study is based on institutional ethical committee guidelines. Since the main group in this research includes the users of Xiaohongshu, the first question on the Questionnaire Star was a screening question. In other words, if the respondent was a user of Xiaohongshu, they would continue to fill in the questionnaire, and if they were not users of Xiaohongshu, they would stop answering the questions directly.

The authors issued the questionnaire through WeChat, China’s leading social network, and personal computers (PCs) simultaneously. The authors used both snowball and convenient sampling approaches to recruit a diverse sample, and respondents filled it out online. There was a 2-yuan reward for the respondents to encourage them to complete the survey. To minimize the sampling bias arising from non-random sampling, the authors do these operations in the back stage of the questionnaire system. The authors (1) set a speed test to eliminate gratuitous or casual responses; (2) asked them to match all answer options to complete the questionnaire or they will not get the reward; (3) added a reminder part, i.e., when response time goes over 25 min, the system will automatically close and regard the survey as invalid; (4) added the same question in the middle of the survey. When the respondents fill in different answers, the authors would regard the questionnaire as invalid questionnaire.

A total of 376 questionnaires were collected. Through screening and rejection of the returned questionnaires, there were 366 valid questionnaires, with an effective rate of 97.34%. The data for the 366 remaining questionnaires were screened according this question: “Do you use the Xiaohongshu application?” 70 respondents replied that they had never used the Xiaohongshu. Therefore, the authors set the sample size to 296 in this study.

### Survey Sample

Among the 296 valid samples, there were 210 female users of Xiaohongshu (70.9%). It shows that women constitute the main user group of Xiaohongshu. The users of Xiaohongshu are 18–25 years old, followed by the 26–30-year-old group. It shows that the users of Xiaohongshu are mainly young people who have a good financial ability. In addition, according to the data from the questionnaire, a large proportion of Xiaohongshu users has an education level of bachelor’s and master’s degrees and above (the range was high school and below, college, bachelor’s and master’s degrees and above), a total of 240 people, or 81.08%. The greatest shopping frequency of users on Xiaohongshu was 0 times (the range was 0 times, 1–2 times, 3–5 times, 6–8 times, and more than 8 times), a total of 112 people, or 37.84%. This phase basically satisfies the hypothesis of the main problem of this research, which indirectly shows that Xiaohongshu users rarely actually shop on Xiaohongshu.

## Data Analysis and Hypothesis Testing

### Reliability and Validity

The authors analyzed the reliability of the questionnaire using SPSS 25.0. The overall sample size (16 items) in the questionnaire scale in this study had a Cronbach’s α of 0.854, greater than 0.8, indicating high reliability. The Cronbach’s α of each variable was also above 0.7, indicating good reliability. Although the Cronbach’s α value of negative reports was lower, the results above 0.6 indicate that the reliability was acceptable. Results are shown in Table 2. Therefore, the five variables in the questionnaire passed the reliability test, which also provided a reliable guarantee for the subsequent data analysis.

**TABLE 2:** Sample reliability analysis.

**Variable**	**Cronbach’s alpha**	**Number of Questions**
Consumer perception of product risk (PPR)	0.799	4
Consumer perception of service risk (PSR)	0.768	3
Degree of trust in other platforms (TOP)	0.702	3
Negative report (NR)	0.626	3
Consumer cross-platform buying behavior (CBB)	0.762	3
Total	0.854	16

For the validity in this study, the authors tested the scale in the questionnaire through structural validity. The data were rotated using the method of maximum variance rotation. Generally speaking, when the Kaiser-Meyer-Olkin (KMO) value is greater than 0.6 and the *p* value obtained by the Bartlett sphericity test is less than 0.05, it can be analyzed by factors. The characteristic root is generally greater than 1 as the standard. When the validity test was performed by factor analysis, the KMO values were 0.784 (perceived product effect risk), 0.690 (perceived service risk), 0.672 (degree of trust in other platforms), 0.624 (negative reporting), and 0.664 (consumer cross-platform purchase behavior); all values were greater than 0.6, and the minimum was 0.624, which indicated that the validity was acceptable, and the maximum was 0.784, which indicated that the validity was good. And Bart’s sphericity test (*p* = 0.000 < 0.05) was passed, which showed that the validity of all variables in this study met the requirements.

### Correlation Analysis and Regression Analysis

Correlation analysis is a common method used to study the closeness statistics between variables, that is, to study the relationship between some quantitative data, such as whether there is a relationship and whether the relationship is close. Descriptive analysis of the degree of closeness is generally performed through correlation coefficients. The authors used Pearson correlation analysis to analyze the relevant variables of this study; see Table 3 for details.

**TABLE 3:** Pearson correlation between variables (*N* = 296).

	**Mean**	**SD**	**PPR**	**PSR**	**TOP**	**NR**	**CBB**
PPR	3.011	0.932	1				
PSR	2.928	0.965	0.637**	1			
TOP	3.280	0.949	0.035	0.154**	1		
NR	3.186	0.879	0.517**	0.563**	0.192**	1	
CBB	3.693	0.985	0.353**	0.375**	0.298**	0.525**	1

Significance level ** *p* < 0.01. CBB, consumer cross-platform buying behavior; NR, negative report; PPR, consumer perception of product risk; PSR, consumer perception of service risk; TOP, degree of trust in other platforms.

As seen from the table, correlation analysis was used to study the correlation between product effects, service risks, trust in other platforms, negative reports, and consumer cross-platform purchase behavior. The outcome shows that the products and services risks, negative reports, and consumer cross-platform buying behavior are 0.637, 0.517, and 0.353, respectively. All were greater than 0, which describes the effect of the products and services risks (*r* = 0.637, *p* < 0.01), negative reports (*r* = 0.517, *p* < 0.01), and consumer cross-platform buying behavior (*r* = 0.353, *p* < 0.01). A total of three items have a positive correlation.

At the same time, the results verify that consumers’ perception of the risk of product effects is related to consumers’ cross-platform buying behavior. Consumer perception of risk services and consumer cross-platform purchase behavior related to discovery and conclusion: the perceived risk of service will affect consumer trust for other platforms.

Regression analysis is a statistical analysis method based on the correlation analysis, which can determine the quantitative relationship between two or more variables (Li, 2018). The authors analyzed the related variables through the analysis method of hierarchical regression. Hierarchical regression is analyzed by dividing into multiple models to study the influence relationship between one variable (classified or quantitative) and another variable (quantitative). In particular, this is used to judge the change of the *R*^2^ value caused by the increase in the degree of trust in other platforms. This paper takes the perceived product effect risk and perceived service risk as independent variables, the degree of trust in other platforms as the intermediate variable, and the consumer’s cross-platform purchase behavior as the dependent variable. The regression analysis was performed on the sample size of the questionnaire, as shown in Table 4.

**TABLE 4:** Results of hierarchical regression analysis between variables.

	**Layer 1**				**Layer 2**			
	***b***	**Standard error**	***t***	***p***	***b***	**Standard error**	***t***	***p***
Constant	2.326**	0.190	12.233	0.000	1.525**	0.244	6.263	0.000
PPR	0.203**	0.073	2.773	0.006	0.233**	0.071	3.286	0.001
PSR	0.258**	0.071	3.638	0.000	0.199**	0.069	2.875	0.004
TOP					0.270**	0.054	4.979	0.000
*R*^2^	0.163				0.228			
*F* value	*F*(2, 293) = 28.433, *p* = 0.000**				*F*(3, 292) = 28.759, *p* = 0.000**			

Dependent Variable (Y): Consumer Cross-Platform Buying Behavior. Significance level **p < 0.01. PPR, consumer perception of product risk; PSR, consumer perception of service risk; TOP, degree of trust in other platforms.

The table shows this hierarchical regression analysis involving Liang models. The independent variables in Model 1 were perceived product risk and perceived service risk. Model 2 added on the basis of Model 1 on the level of trust in other platforms; Models 1 and 2 of the dependent variable were cross-platform consumer behavior. In Model 1, a linear regression analysis using perceived product effect risk and perceived service risk as independent variables and consumer cross-platform purchase behavior as the dependent variable obtained the model *R*^2^ value of 0.163, which means that perceived product effect risk and perceived service risk can explain the 16.3% change in consumer cross-platform buying behavior. Therefore, the model formula is: Consumer cross-platform purchase behavior = 2.326 + 0.203 × Product effect + 0.258 × Service risk. In addition, the regression coefficient value of the product effect is 0.203 (*t* = 2.773, *p* < 0.01), which means that the perceived product effect risk will have a significant positive impact on consumers’ cross-platform buying behavior. The regression coefficient value of perceived service risk is 0.258 (*t* = 3.638, *p* < 0.01), which means that the perceived service risk will also have a significant positive impact on consumers’ cross-platform purchasing behavior. A summary of the analysis shows that the perceived risk of product performance and risk-aware services on consumers’ cross-platform buying behavior will have a significant positive effect on relations; that is, if consumers perceive the effect of risks of products and services on the Xiaohongshu platform, then they are more likely to choose to purchase target products across platforms.

For Model 2 terms, it was added on the basis of Model 1 on the level of trust variables for other platforms (such as Taobao, Jingdong, Sea Amoy, and Acquaintance purchasing), the *F* value showed significant changes (*p* < 0.05), meaning that the degree of trust in other platforms (such as Taobao, Jingdong, Haitao, and Acquaintance purchasing) adds an explanatory meaning to the model. In addition, the *R*^2^ value rose from 0.163 to 0.228, which means that the degree of trust in other platforms can explain 6.6% of consumers’ cross-platform purchasing behavior. Specifically, the trust coefficient in relation to these other platforms is 0.270, and it exhibits significance (*t* = 4.979, *p* < 0.01), meaning that for other platforms, it means that the level of trust related to other platforms has a significant positive influence on consumer across-platform buying behavior.

In short, the above analysis further validates H1: The risk of product effects that consumers perceive has a positive effect on consumers’ cross-platform buying behavior. In other words, if consumers are more aware of the risks of product effects on the Xiaohongshu platform, then the more likely they are to choose a cross-platform purchase. H2: Consumers’ perception of the risk of services has a positive impact on their cross-platform purchase behavior. In other words, the more the consumer perceives a risk of services on the Xiaohongshu platform, the more likely he or she is to choose cross-platform purchases. The results also showed that the level of trust in other platforms will have a significant positive relationship to influence with respect to consumer buying across platforms. In other words, the higher the level of trust in other platforms, the more likely that Xiaohongshu users will buy cross-platform target products.

### Moderating Effects and Hypothesis Testing

Moderating effects were studied mainly to learn the effects of negative reports on the moderating variable and to learn whether the magnitude of the effect of the independent variable on the dependent variable was different in different situations. This study verifies whether negative reports have a moderating effect. Since the variables in this study consist of quantitative data, it is necessary to centralize or standardize the two latitudes of perceived risk of the independent variables and the negative reports of the adjusted variables. By a centralized processing method, the specific operation method is to first measure the average value of each variable and then subtract the average value of each variable to obtain the required values and then multiply these values to sort out each variable; second, people will get the interaction terms, and then people can get the formula of the regression equation of consumers’ cross-platform buy behavior for the independent variable perception of risk and the negative report of the adjustment variable to get the measurement coefficients of each variable. Finally, the consumer’s cross-platform buying behavior for the independent variable’s perception of product effect risk was analyzed. Perceived service risk and moderator negative reports do cross terms back to go get a new coefficient of determination. The following (Table 5) are the results of the data analysis of the impact of negative reports on moderator variables with respect to perceived product risk and perceived service risk effects on the consumer’s cross-platform purchase behavior of dependent variables.

**TABLE 5:** Analysis of regulatory effect results–perceived risk of product and service effects.

	**Perceived product risk effect**			**Perceived service risk effect**		
	**Model 1**	**Model 2**	**Model 3**	**Model 1**	**Model 2**	**Model 3**
Constant	3.693 (68.831**)	3.693 (76.037**)	3.741 (72.216**)	3.693 (69.463**)	3.693 (76.042**)	3.783 (73.490**)
PPR/PSR effect	0.373 (6.472**)	0.118 (1.929)	0.097 (1.597)	0.383 (6.934**)	0.118 (1.941)	0.114 (1.926)
NR		0.524 (8.110**)	0.490 (7.480**)		0.515 (7.703**)	0.429 (6.318**)
PPR/PSR effect* NR			0.114 (−2.512*)			0.189 (−4.356**)
*R*^2^	0.125	0.285	0.300	0.141	0.285	0.329
*F* value	*F*(1, 294) = 41.889, *p* = 0.000**	*F*(2, 293) = 58.445, *p* = 0.000 **	*F*(3, 292) = 41.774, *p* = 0.000**	*F*(1, 294) = 48.084, *p* = 0.000**	*F*(2, 293) = 58.476, *p* = 0.000**	*F*(3, 292) = 47.703, *p* = 0.000**

Dependent Variable (Y): Consumer Cross-Platform Buying Behavior. Significance level * *p* < 0.05 ** *p* < 0.01 The *t* value is in parentheses (). NR, negative report; PPR, consumer perception of product risk; PSR, consumer perception of service risk.

According to the above table, the analysis of the adjustment effect research is divided into three models. Model 1 analyses the influence of the independent variable, perception of product effect risk, on consumers’ cross-platform purchase behavior. Model 2 adds the adjustment variables based on Model 1, negative reports. Model 3 includes the independent variables on the basis of Model 2 on the perceived product performance risks, and it regulates the interaction term variable, negative reports. If the *F* value from Model 2 to Model 3 changes significantly, it represents the presence of a regulatory effect, and if the Model 3 interaction term is significant, it also means that there is a regulatory effect. As can be seen from the table, the effects of the perceived risk of the product on the independent variables were significant (β = 0.373, *p* < 0.05). It has been verified once again that the perceived product effect risk has a significant impact on consumers’ cross-platform purchasing behavior. In addition, in Model 3, the interaction term between perceived product effect risk and negative reporting appears significant (β = 0.114, *p* < 0.05). This means that the perceived product effect risks have a significant impact on the cross-platform buying behavior, and negative reports at different levels have a significant impact on different magnitudes. In other words, negative reports play a moderating role in consumers’ perception of the risk of product effects on Xiaohongshu and on cross-platform buying behaviors. The more negative reports there are, the more the relationship between the two is promoted.

A clear table, perceived risk service presents a significant outcome (β = 0.383, *p* < 0.05). It has been verified once again that the perceived service risk has a significant impact on consumers’ cross-platform purchasing behavior. In addition, in Model 3, the interaction term between perceived service risk and negative reporting is significant (β = 0.189, *p* < 0.05). This means that the perceived risk of service for consumers have a significant impact on consumer’s across-platforms buying behavior. Negative reports manipulated variables at different levels, the impact of the magnitude of significant differences. In other words, with the perceived risk of negative reports for consumer services across Xiaohongshu, the relationship with platform buying behavior plays a moderating role. When there are more negative reports, the relationship between the two is promoted.

### Intermediary Effects and Hypothesis Testing

The authors conducted a bootstrap test with Model 5 in the SPSS 25.0 plug-in process to verify the existence of the mediation effect. The results are shown in Table 6.

**TABLE 6:** Test of the mediating effect of H3 and H4^[1]^.

**Mediation path**	**Indirect effect**	**Boot standard error**	**95% Confidence interval**	**Conclusion**
X1–M1–Y	0.0503	0.1740	0.0811 0.2782	Significant
X2–M1–Y	0.0515	0.1719	0.0780 0.2816	Significant

*^[1]^X1 is the perceived product effect risk, X2 is the perceived service risk, M1 is the degree of trust in other platforms (such as Taobao, Jingdong, Haitao, and Acquaintance purchasing), and Y is the consumer’s cross-platform purchase behavior.*

The authors used Preacher and Hayes (2008) method of testing a proposed intermediary. The authors used the perceived risk and the perceived effects of products and services for risk as independent variables, used trust in other platforms as the mediator, and used consumer buying behavior across platforms as the dependent variable to build a model, with a 95% confidence interval and 5,000 sampling times. The displayed perceived product effect risk (*b* = 0.1740, SE = 0.0503, 95% confidence interval: 0.0811, 0.2782) and the perceived service risk (*b* = 0.1719, SE = 0.0515, 95% confidence interval 0.0780, 0.2816) for cross-platform consumer buying behavior with respect to the degree of trust in other platforms showed a significant indirect effect. This means that trust plays an intermediary effect, and therefore, H3 and H4 are supported. In other words, the more strongly consumers perceive product effects and service risks on the Xiaohongshu platform, the more trust they have in other platforms, and the more likely they are to choose to purchase target products across platforms.

### Research Results and Findings

This section summarizes the research hypotheses based on the above analysis results, and a breakdown of the findings is as follows: The risk perception of product effects as well as service for consumers has a positive impact on the cross-platform purchase behavior. Consumers’ level of trust in other platforms has important potential in the buying behavior of consumers across platforms insofar as consumers’ perceived risk and perceived service product performance risks will affect consumption; The level of trust that consumers have in other platforms further affects consumers’ cross-platform buying behavior; Negative reports also play a role in consumer cross-platform buying behavior. Perceived product effect risk and perceived service risk will affect consumers’ trust in other platforms and will further affect consumers’ cross-platform buying behavior.

## Research Conclusion and Discussion

### Research Conclusions

With the rapid development of the Internet, cross-border e-commerce has also emerged, with a growing number of products and a growing number of netizens. As a new app platform, Xiaohongshu not only provides Internet users with a variety of strategies for eating, drinking, and playing but also has certain options for consumers to shop. But from long-term observation, the authors have found that although consumers have accepted the functions provided by Xiaohongshu, they have not used it as a shopping platform. The aim of this article therefore is to provide some reference suggestions for cross-border e-commerce platforms like Xiaohongshu by analyzing the relationship between consumers’ perceived risk and consumer cross-platform buying behavior.

In this paper, the risks of consumer perception of product effects and perceived service risks are taken as independent variables, the degree of trust in other platforms is used as an intermediate variable, negative reports are used as moderating variables, and the influence of consumers’ cross-platform purchase behavior is used as a dependent variable. Suppose that consumers’ cross-platform purchasing behavior is determined by these variables. The results show that consumers’ perception of product effectiveness risks and perceived service risks, their level of trust in other platforms, and negative reports play an important role in consumers’ cross-platform buying behavior. First of all, the risks of consumers’ perception of product effects and consumer cross-platform buying behavior as well as the risks of consumer perception of services and consumer cross-platform buying behavior are significantly positively correlated. Secondly, consumers’ level of trust in other platforms (such as Taobao, Jingdong, Sea Amoy, and Acquaintance purchasing) has important potential in the buying behavior of consumers across platforms; consumers’ perceived risk and perceived service product performance risks will affect consumption. The level of trust that consumers have in other platforms further affects consumers’ cross-platform buying behavior. Finally, negative reports also play a role in consumer cross-platform buying behavior. Perceived product effect risk and perceived service risk will affect consumers’ trust in other platforms and will further affect consumers’ cross-platform buying behavior. However, this influence is positive when there are many negative reports and negative when there are few negative reports.

### Discussion

From the analysis results above, the risks of consumers’ perception of product effects and risks of perceived services will positively affect consumers’ purchase behavior across platforms. Therefore, the authors believe that the merchants on the platform should strictly control and screen products, strictly implement national policies and regulations, do a good job in word-of-mouth publicity while ensuring product quality, establish a good corporate image, and provide consumers with more positive information. This will allow consumers to reduce the frequency of cross-platform purchases. In particular, it is possible to strengthen strict checks on the entry of merchants, for example, in establishing a detailed evaluation mechanism and requiring merchants to provide relevant credit information as one of the evaluation criteria. It is also necessary to do follow-up reviews of already settled merchants to ensure product quality and avoid fakes flowing into the market as much as possible. At the same time, there must be a certain response strategy for some illegal operations of the settled merchants, such as billing, to ensure the merchants’ quality. Both the service level of the platform and the service level of the resident merchants should also be taken very seriously. For example, for resident merchants, a series of assessment mechanisms can be established to strengthen the consciousness and initiative of employees. The negative evaluation of some products should be handled positively to be recognized by consumers. As for the platform, it should pay attention to the construction of service quality; in particular, the service personnel of the platform should answer consumers’ questions as quickly and effectively as possible.

Secondly, from the analysis results above, the risks of perceived product effects and perceived service risks will affect consumers’ trust in other platforms (such as Taobao, Jingdong, Haitao, and Acquaintance purchasing) and will further affect consumers’ cross-platform buying behavior. Therefore, the authors believe that Xiaohongshu should strengthen the ways they relate to consumers’ psychology. The way to make consumers trust the platform is to gain their trust and recognition of the platform by enhancing their “hard power” and “soft power.” The “hard power” of the platform can be enhanced through the security of transactions, the credibility of the merchants, the technical advantages of the platform, and the number of users. The “soft power” of the platform can be enhanced by enhancing the corporate culture and at the same time paying attention to the construction and management of their credibility. The increase in consumers’ trust in this platform will naturally affect consumers’ purchasing decisions.

Finally, perceived product risk and perceived service risk will affect consumers’ trust in other platforms (such as Taobao, Jingdong, Haitao, and Acquaintance purchasing) and will further affect consumers’ cross-platform buying behavior. However, this influence is positive when there are many negative reports and negative when there are few negative reports. Therefore, the platform side should always pay attention to relevant reports in the news media to be able to actively respond and adjust when negative reports occur. When negative media reports do appear, the issue should not be viewed in a one-sided manner, and crisis public relations should be adopted in a reasonable and effective manner.

## Strengths and Limitations

The strengths of the work are listed as follows: This is an interesting topic and in keeping with academic conversations around risk and trust in mobile apps. And the findings are interesting and do advance this area of understanding.

The limitations of the work are listed as follows: This study selected the two dimensions of consumer perception of product risk and perceived service risk as independent variables of perceived risk, but these two dimensions cannot represent all dimensions of consumer perceived risk, such as perceived risk, social risk, time risk, physical injury risk, etc. In fact, there are various factors that affect consumers’ cross-platform buying behaviors, such as consumers’ usage habits for a certain type of app, consumers’ perceived value of products, and consumers’ personal likes and dislikes. However, given the authors’ own research capabilities and the operability and feasibility of the investigation, this paper selected consumers’ perceived product effect risk and perceived service risk as two dimensions of the independent variable, perceived risk, and at the same time selected trust as an intermediate variable and negative reporting as a moderating variable. It is hoped that in future research, more dimensions of the perceived risk of independent variables can be tapped for further research and exploration. In addition, the literature review is missing several more contemporary research in mobile commerce, mobile shopping, and mobile apps that concern perceived risks. The data analysis and approach are required to be more heavily justified, and some missing tests must be included. A more in-depth analysis using some SEM methods would heighten the rigor of the findings.

This study distributed questionnaires through online channels, which inevitably produced some potential disadvantages, such as the possibility of deceptiveness, acquaintances filling out the questionnaires, and the inability to protect personal information. In this study, a total of 366 samples were collected; although this samples size meets the requirements of academic research, it is still small. The sample may not be representative and may have some impact on the results of subsequent studies. Therefore, the authors expect to expand the sample size and collect much more extensive data in future research for increased persuasiveness.

## Data Availability Statement

The raw data supporting the conclusions of this article will be made available by the authors, without undue reservation.

## Ethics Statement

The studies involving human participants were reviewed and approved by Shenzhen University Ethics Committee. The patients/participants provided their written informed consent to participate in this study. Written informed consent was obtained from the individual(s) for the publication of any potentially identifiable images or data included in this article.

## Author Contributions

All authors listed have made a substantial, direct and intellectual contribution to the work, and approved it for publication.

## Conflict of Interest

The authors declare that the research was conducted in the absence of any commercial or financial relationships that could be construed as a potential conflict of interest.
